# Deep learning or radiomics based on CT for predicting the response of gastric cancer to neoadjuvant chemotherapy: a meta-analysis and systematic review

**DOI:** 10.3389/fonc.2024.1363812

**Published:** 2024-03-27

**Authors:** Zhixian Bao, Jie Du, Ya Zheng, Qinghong Guo, Rui Ji

**Affiliations:** ^1^ Department of Gastroenterology, the First Hospital of Lanzhou University, Lanzhou, China; ^2^ Department of Gastroenterology, Xi’an NO.1 Hospital, Xi’an, Shaanxi, China; ^3^ Department of Social Medicine and Health Management, School of Public Health, Lanzhou University, Lanzhou, China; ^4^ Gansu Province Clinical Research Center for Digestive Diseases, The First Hospital of Lanzhou University, Lanzhou, China

**Keywords:** gastric cancer, neoadjuvant chemotherapy, deep learning, radiomics, artificial intelligence, meta-analysis

## Abstract

**Background:**

Artificial intelligence (AI) models, clinical models (CM), and the integrated model (IM) are utilized to evaluate the response to neoadjuvant chemotherapy (NACT) in patients diagnosed with gastric cancer.

**Objective:**

The objective is to identify the diagnostic test of the AI model and to compare the accuracy of AI, CM, and IM through a comprehensive summary of head-to-head comparative studies.

**Methods:**

PubMed, Web of Science, Cochrane Library, and Embase were systematically searched until September 5, 2023, to compile English language studies without regional restrictions. The quality of the included studies was evaluated using the Quality Assessment of Diagnostic Accuracy Studies-2 (QUADAS-2) criteria. Forest plots were utilized to illustrate the findings of diagnostic accuracy, while Hierarchical Summary Receiver Operating Characteristic curves were generated to estimate sensitivity (SEN) and specificity (SPE). Meta-regression was applied to analyze heterogeneity across the studies. To assess the presence of publication bias, Deeks’ funnel plot and an asymmetry test were employed.

**Results:**

A total of 9 studies, comprising 3313 patients, were included for the AI model, with 7 head-to-head comparative studies involving 2699 patients. Across the 9 studies, the pooled SEN for the AI model was 0.75 (95% confidence interval (CI): 0.66, 0.82), and SPE was 0.77 (95% CI: 0.69, 0.84). Meta-regression was conducted, revealing that the cut-off value, approach to predicting response, and gold standard might be sources of heterogeneity. In the head-to-head comparative studies, the pooled SEN for AI was 0.77 (95% CI: 0.69, 0.84) with SPE at 0.79 (95% CI: 0.70, 0.85). For CM, the pooled SEN was 0.67 (95% CI: 0.57, 0.77) with SPE at 0.59 (95% CI: 0.54, 0.64), while for IM, the pooled SEN was 0.83 (95% CI: 0.79, 0.86) with SPE at 0.69 (95% CI: 0.56, 0.79). Notably, there was no statistical difference, except that IM exhibited higher SEN than AI, while maintaining a similar level of SPE in pairwise comparisons. In the Receiver Operating Characteristic analysis subgroup, the CT-based Deep Learning (DL) subgroup, and the National Comprehensive Cancer Network (NCCN) guideline subgroup, the AI model exhibited higher SEN but lower SPE compared to the IM. Conversely, in the training cohort subgroup and the internal validation cohort subgroup, the AI model demonstrated lower SEN but higher SPE than the IM. The subgroup analysis underscored that factors such as the number of cohorts, cohort type, cut-off value, approach to predicting response, and choice of gold standard could impact the reliability and robustness of the results.

**Conclusion:**

AI has demonstrated its viability as a tool for predicting the response of GC patients to NACT Furthermore, CT-based DL model in AI was sensitive to extract tumor features and predict the response. The results of subgroup analysis also supported the above conclusions. Large-scale rigorously designed diagnostic accuracy studies and head-to-head comparative studies are anticipated.

**Systematic review registration:**

PROSPERO, CRD42022377030.

## 1 Introduction

Gastric cancer (GC) stands as the second leading cause of cancer-related deaths globally, positioning it among the most prevalent malignant tumors in Asia (1, 2). In 2018 alone, there were over 1 million new cases of GC, resulting in approximately 784,000 deaths worldwide (1). Surgery remains the primary treatment for locally advanced GC, yet the 5-year survival rate post curative resection hovers between 20% to 30% (3). Unfortunately, the majority of patients (80-90%) are diagnosed at advanced stages (4, 5). In China, the prognosis for patients with locally advanced GC is particularly poor (6, 7).

In recent years, neoadjuvant chemotherapy (NACT) has demonstrated effectiveness in treating GC patients, exhibiting potential to enhance prognosis and elevate the 5-year survival rate to over 35% (8, 9). Nevertheless, NACT carries certain limitations, including toxic reactions in patients (10–13), suboptimal responses in some cases, missed treatment opportunities, and nearly 30% of patients developing resistance to chemotherapy (14). The evaluation of a patient’s response to NACT currently relies on invasive histopathological tests conducted post-surgery, providing limited guidance for clinical practice.

Computed tomography (CT) is widely employed for assessing the response to NACT in GC patients. However, the current method of extracting image features through visual assessment or quantitative imaging parameters is deemed unreliable. In contrast, radiomics emerges as a rapidly evolving tool that predicts the response of GC patients to NACT by analyzing high-throughput quantitative images and extracting effective prognostic features. Accurate delineation of the tumor is crucial for feature extraction and model building, but limitations arise from the variability in physician experience, impacting the empirical results of tumor delineation.The combination of radiomics with clinical features has demonstrated outstanding performance in predicting the response to NACT. DL, an emerging approach rooted in artificial intelligence, autonomously learns key disease features from clinical images and extracts accurate features relevant to specific needs (15–18). DL has showcased superior performance in capturing tumor features and predicting prognosis across various cancer types (19–24).

Several studies have explored the utility of CT-based DL and CT-based radiomics in predicting the response to NACT in GC patients. However, these studies have produced inconsistent or conflicting results concerning diagnostic accuracy when compared to the gold standard. Additionally, these studies have reported the diagnostic accuracy of clinical models (CM) and integrated models (IM) in reference to the gold standard. Notably, there is currently a lack of reviews analyzing the diagnostic accuracy of DL and radiomics in predicting the response to NACT in GC patients. Therefore, this meta-analysis was conducted to offer a comprehensive analysis of the available literature.

## 2 Methods

### 2.1 Protocol

The protocol was registered on the International Prospective Register of Systematic Reviews (PROSPERO) (CRD42022377030) and performed according to Preferred Reporting Items for Systemic Reviews and Meta-Analysis (PRISMA) guidelines (25).

### 2.2 Search strategy

The following search interests were considered when constructing the strategy: (1) terms related to GC; (2) terms related to AI; (3) terms related to neoadjuvant therapy. The online databases PubMed, Web of Science, Cochrane Library, and Embase were searched prior to September 5, 2023. Additionally, during the full-text review phase, the reference lists of all included articles and retained systematic reviews were manually screened to identify any studies that may have been missed in the initial search. Free words adjusted by the different databases and theme words were combined to form the search. The search strategies used for PubMed can be found in the **Supplementary Material**.

### 2.3 Eligibility criteria

Only studies that met the following criteria were included in this analysis: (a) the patient population consisted of individuals with histologically (biopsy-) confirmed gastric adenocarcinoma; (b) CT scans were performed prior to neoadjuvant chemotherapy; (c) an AI algorithm was used to predict the response of GC; (d) a reference standard was available and reported in detail; (e) the study data could be extracted and organized into a standard 2×2 table; and (f) the study design was either a comparative study or a randomized controlled trial. Any studies that met the following exclusion criteria were not considered: (a) studies with duplicate data (only the study with the most comprehensive data was selected); (b) case reports, letters, reviews, comments, meeting records, or protocol studies; (c) animal studies; or (d) publications on diseases other than GC.

### 2.4 Study selection

Two reviewers, Du and Bao, independently selected studies. During the title and abstract review phase, all potentially relevant studies were retrieved. Then, the full texts were reviewed based on inclusion and exclusion criteria. Any discrepancies were resolved through mutual discussion until a consensus was reached or by involving a third author (Zheng) who was kept blind to the study details.

### 2.5 Quality assessment

The Quality Assessment of Diagnostic Accuracy Studies-2 (QUADAS-2) tool was utilized to evaluate the methodological quality of the included articles. This tool consists of four domains: patient selection, index test, reference standard, and flow and timing. Each domain was assessed for high, low, or unclear risk of bias, and the first three domains were also evaluated for high, low, or unclear concerns regarding applicability (26). To generate the summary figure of the methodological quality evaluation, Review Manager version 5.4.1 (Review Manager for Windows 7, Nordic Cochrane Centre) was employed.

### 2.6 Data extraction

The data were collected and independently verified by two reviewers (Du, Bao). Any discrepancies were resolved through mutual discussion until a consensus was reached or by involving a third author (Zheng) who was kept blind to the study details. The relevant data included the following: (a) baseline characteristics such as title, first author, publication year, region, study design, sample size, cohorts, pathological and clinical type, diagnosis method, type of CT, CT phase, and NAC regimen; (b) diagnostic accuracy information, including the gold standard, cut-off value, type of AI algorithm, the number of good response (GR) or no-GR to NAC, and diagnostic performance indices of the AI algorithm, CM, and IM (which include sensitivity (SEN), specificity (SPE), positive predictive value (PPV), negative predictive value (NPV), positive likelihood ratio (PLR), negative likelihood ratio (NLR), diagnostic odds ratio (DOR), or accuracy); and (c) methodological evaluation information. Some studies have multiple cohorts, so before conducting the meta-analysis, the standard 2×2 tables of all cohorts in each article need to be consolidated.

### 2.7 Data synthesis and analysis

Forest plots were used to analyze the diagnostic accuracy of each test, along with a 95% confidence interval (CI), using Stata 14 (Stata Corporation, College Station, TX, USA). Hierarchical summary receiver operating characteristic (HSROC) curves were constructed to estimate and compare SEN and SPE. Meta-regression was applied to assess the source of heterogeneity. To evaluate the presence of publication bias, Deeks’ funnel plot and an asymmetry test were used. Statistical significance was indicated by a p-value < 0.05. Heterogeneity was evaluated using I^2^ statistics and standard χ^2^-testing, where I^2^ > 50% or p < 0.05 indicated notable heterogeneity. The test performance was computed using a random-effects coefficient binary regression model, unless a fixed-effects coefficient binary regression model was applicable (27).

Subgroup analysis was performed as follows: (a) cut-off value (determined by analyzing Receiver Operating Characteristic (ROC) or not reported); (b) location of cancer (GC or esophagogastric junction cancer); (c) number of cohorts (1 or more than 1); (d) type of cohorts (training, internal validation, or external validation); (e) approach of predicting response (CT-based deep learning or CT-based radiomics); (f) gold standard (National Comprehensive Cancer Network (NCCN) guidelines or others). Generally, diagnostic accuracy was higher in the training cohort compared to the validation cohort, particularly the external validation cohort. We conducted pairwise comparisons among the three cohorts to assess whether the test performance varied across cohorts. The presence of publication bias was assessed using Deeks’ funnel plot and an asymmetry test (28).

## 3 Results

### 3.1 Study selection

**Figure 1:**
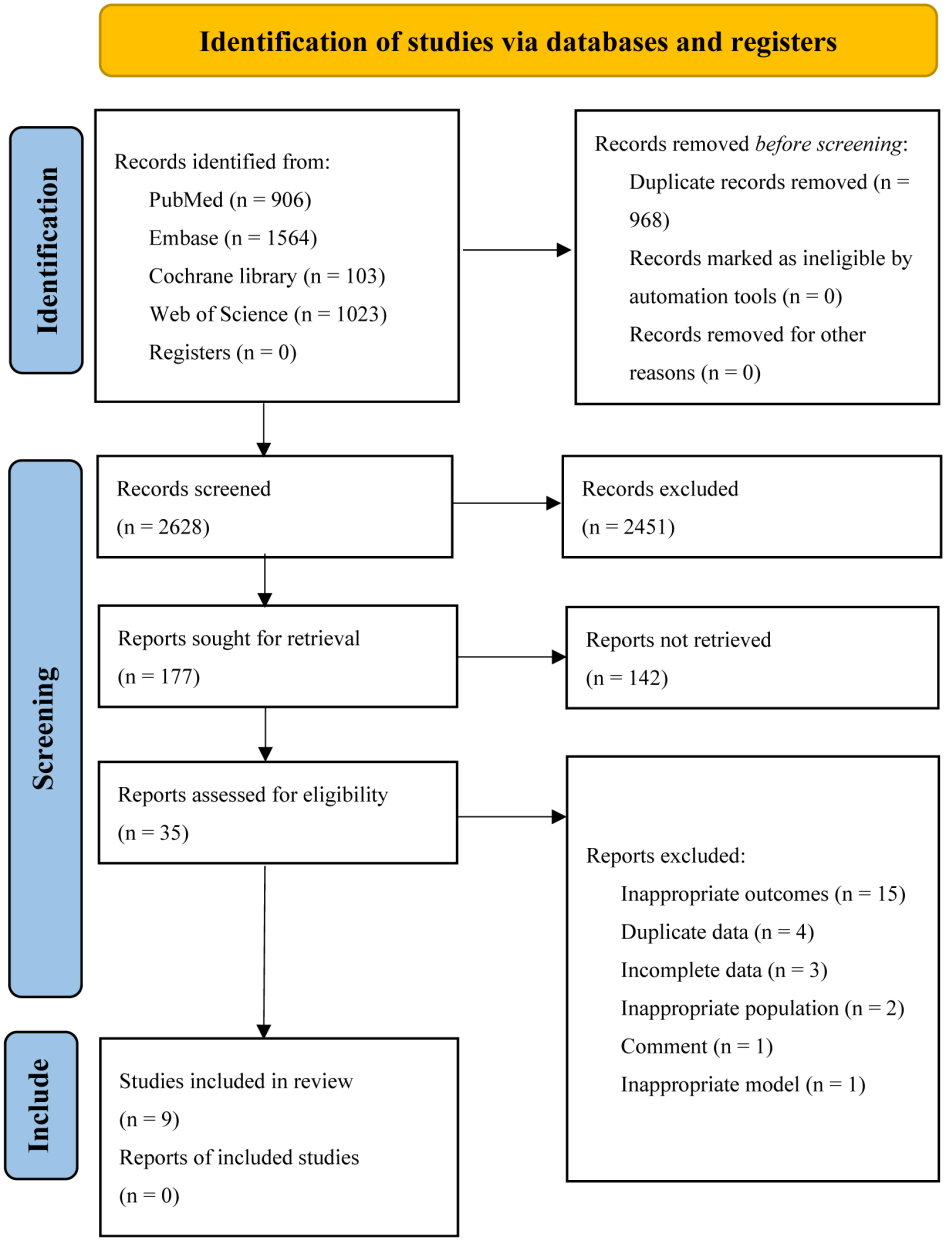
Flow diagram of the selection process for the studies.

Five databases were searched, resulting in a total of 3596 articles. The EndNote software was then used to remove duplicates, leaving 2628 articles. After screening the titles and abstracts, 177 studies were selected for full-text reading. Finally, 9 studies, comprising 23 cohorts, were included for assessing the diagnostic accuracy of AI algorithms in predicting the response of GC to neoadjuvant chemotherapy (29–37). Out of these, 7 head-to-head studies were analyzed to compare the diagnostic accuracy of AI, CM, and IM. Please refer to **Figure 1** for the flowchart depicting the screening process.

### 3.2 Study characteristics

**Figure 2:**
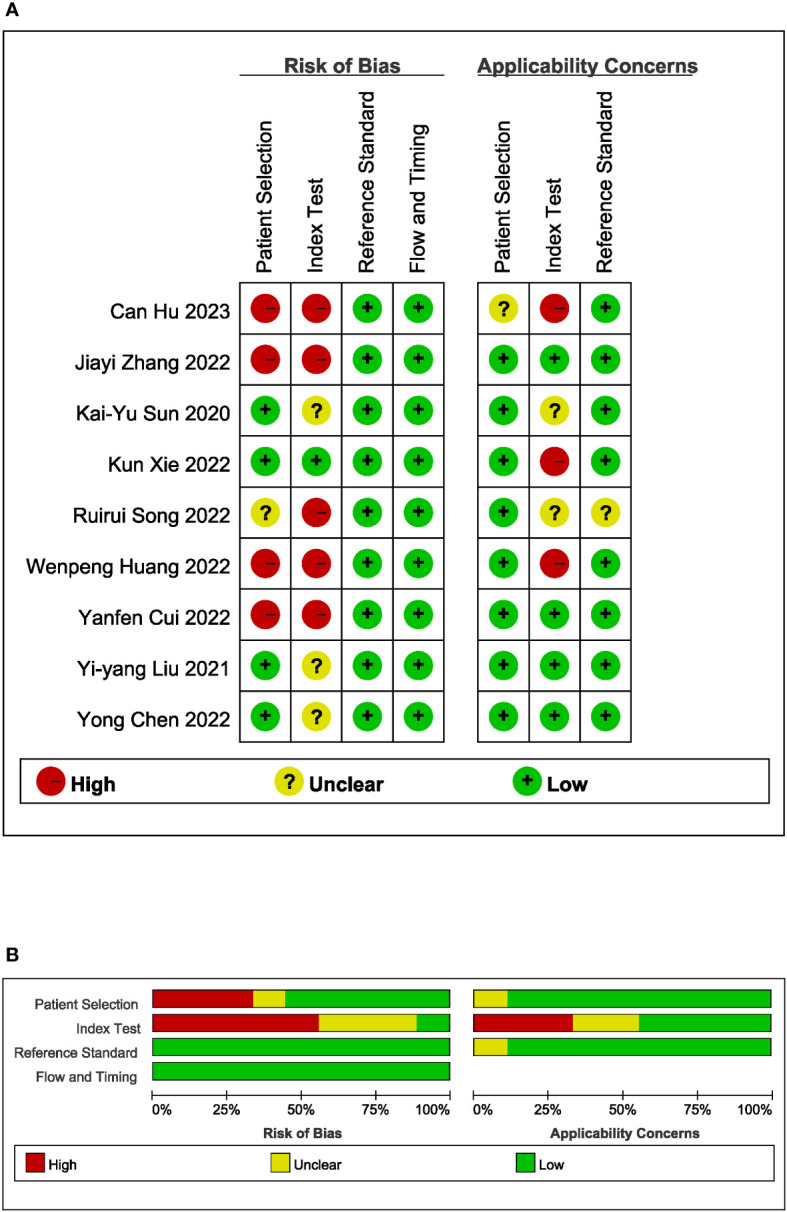
Methodological quality of all 9 included studies. **(A)** quality summary; **(B)** quality graph.

All studies were conducted in Asia, with one study from Japan and the remaining studies originating from China. All studies were retrospective in nature. A total of seven head-to-head comparative studies were included, involving 2709 patients. The pathological types of all studies were adenocarcinomas. The AI algorithms used in these studies included convolutional neural networks, support vector machine, extremely randomized tree, the least absolute shrinkage and selection operator, random forest, naive Bayes, logistic regression, and extreme gradient boosting. The response of GC to neoadjuvant chemotherapy was determined using the NCCA as the gold standard in five studies, Tumor Regression Grading in two studies, Response Evaluation Criteria in Solid Tumors in one study, and reference to other literature as the gold standard in one study. The quality assessment of the nine studies was rated as moderate. **Table 1** and **Figure 2** provide an overview of the characteristics of the included studies and the quality evaluation.

**Table 1:** Characteristics of included studies.

Study	Country	Patients		Pathological type	Diagnosis	Stage of cancer (No./%)	Cut-off value	Type of AI	Approach of predict response	Type of CT	CT phase	Gold standard	NACT regimen
		Male	Female										
Jiayi Zhang 2022	China	302	89	locally advanced gastric adenocarcinoma	histopathologically confirmed	T2 (15, 3.83%); T3 (160, 40.92%); T4a (182, 46.55%); T4b (34, 8.70%); N0 (54, 13.81%); N1 (109, 27.88%); N2(129, 32.99%); N3(99, 25.32%)	ROC analysis	CNNs	CT-based deep learning	CT	portal venous phase	NCCN guideline (v. 4)	XELOX, SOX, FOLFOX
Kai-Yu Sun 2020	China	18	14	gastric adenocarcinoma	histologically confirmed	T3 (2, 9.38%); T4 (29, 90.62%); N0 (1, 3.13%); N1-3 (31, 96.87%); M0 (19, 59.38%); M1 (13, 40.62%)	NR	SVM/Extra-Trees	CT-based radiomics	contrast-enhanced multidetector CT	venous phase	Mandard TRG system	SOX
Yi-yang Liu 2021	Japan	48	21	locally advanced gastric adenocarcinoma	histologically confirmed	N0 (11, 15.94%); N1 (27, 39.13%); N2 (16, 23.19%); N3 (15, 21.74%)	NR	LASSO	CT-based radiomics	dual-energy CT	venous phase	RECIST (v. 1.1)	XELOX, SOX
Can Hu 2023	China	785	275	locally advanced gastric/esophagogastric junction adenocarcinoma or others	histologically confirmed	T1-2 (75, 7.08%); T3-4 (985, 92.92%); N0-1 (213, 20.09%); N2-3 (847, 79.91%); M0 (921, 86.89%); M1 (139, 13.11%)	NR	CNNs	CT-based deep learning	CT/MRI	portal venous phase	NCCN guideline (v. 3)	FOLFOX
Yong Chen 2022	China	233	113	gastric adenocarcinoma	histopathologically confirmed	T2 (6, 1.73%); T3 (23, 6.65%); T4 (327, 91.62%); N0 (7, 2.02%); N1 (64, 18.50%); N2(161, 46.53%); N3(114, 32.95%)	NR	RF	CT-based radiomics	contrast-enhanced CT	portalvenous and delayed phases	Four-grade criterion	EOX, SOX, FLOT, XELOX, FOLFOX
Yanfen Cui 2022	China	552	167	locally advanced gastric adenocarcinoma	histologically confirmed	T2 (21, 2.92%); T3 (275, 38.25%); T4a (364, 50.63%); T4b (59, 8.21%); N0 (80, 11.13%); N1 (109, 15.16%); N2(205, 28.51%); N3(192, 26.70%)	ROC analysis	CNNs	CT-based deep learning	contrast-enhanced CT	arterial and venous phases	NCCN guideline (v. 4)	SOX
Wenpeng Huang 2022	China	73	19	advanced esophagogastric junction adenocarcinoma	histopathologically confirmed	T2-3 (40, 43.48%); T4 (52, 56.52%); N0-1 (55, 59.78%); N2-3 (37, 40.22%)	NR	naive Bayes	CT-based radiomics	contrast-enhanced CT	arterial and venous phases	TRG system	SOX, XELOX
Kun Xie 2022	China	92	32	locally advanced gastric adenocarcinoma	histopathologically confirmed	T2-3 (61, 49.19%); T4 (63, 50.81%); N0 (18, 14.52%); N1 (28, 22.58%); N2(39, 31.45%); N3(39, 31.45%)	NR	SVM	CT-based radiomics	contrast-enhanced CT	portal venous phase	NCCN guideline (v. 4)	SOX
Ruirui Song 2022	China	372	118	locally advanced gastric adenocarcinoma	histopathologically confirmed	T2 (12, 2.45%); T3 (151, 30.82%); T4a (285, 58.16%); T4b (42, 8.57%); N0 (60, 12.24%); N1 (122, 24.90%); N2(154, 31.43%); N3a(118, 24.08%); N3b(36, 7.35%)	ROC analysis	LR/RF/SVM/XGB	CT-based radiomics	contrast-enhanced CT	arterial and portal venous phases	NCCN guideline	SOX, FOLT, XELOX

CT, computed tomography; AI, artificial intelligence; NACT, Neoadjuvant chemotherapy; NCCN, National Comprehensive Cancer Network; NR, not reported; TRG, tumour regression grading; RECIST, Response Evaluation Criteria in Solid Tumors; CNNs, convolutional neural networks; SVM, support vector machine; Extra-Trees, Extremely Randomised Trees; LASSO, the least absolute shrinkage and selection operator; LR, logistic regression; RF, random forest; XGB, extreme gradient boosting; MRI, magnetic resonance imaging; XELOX, oxaliplatin and capecitabine; SOX, S-1 and oxaliplatin; FOLFOX, oxaliplatin, folinic acid and 5-fluorouracil; EOX, epirubicin, oxaliplatin, and capecitabine; FLOT, 5-fluorouracil, leucovorin, docetaxel and oxaliplatin.

### 3.3 Diagnostic test accuracy of AI algorithm

**Figure 3:**
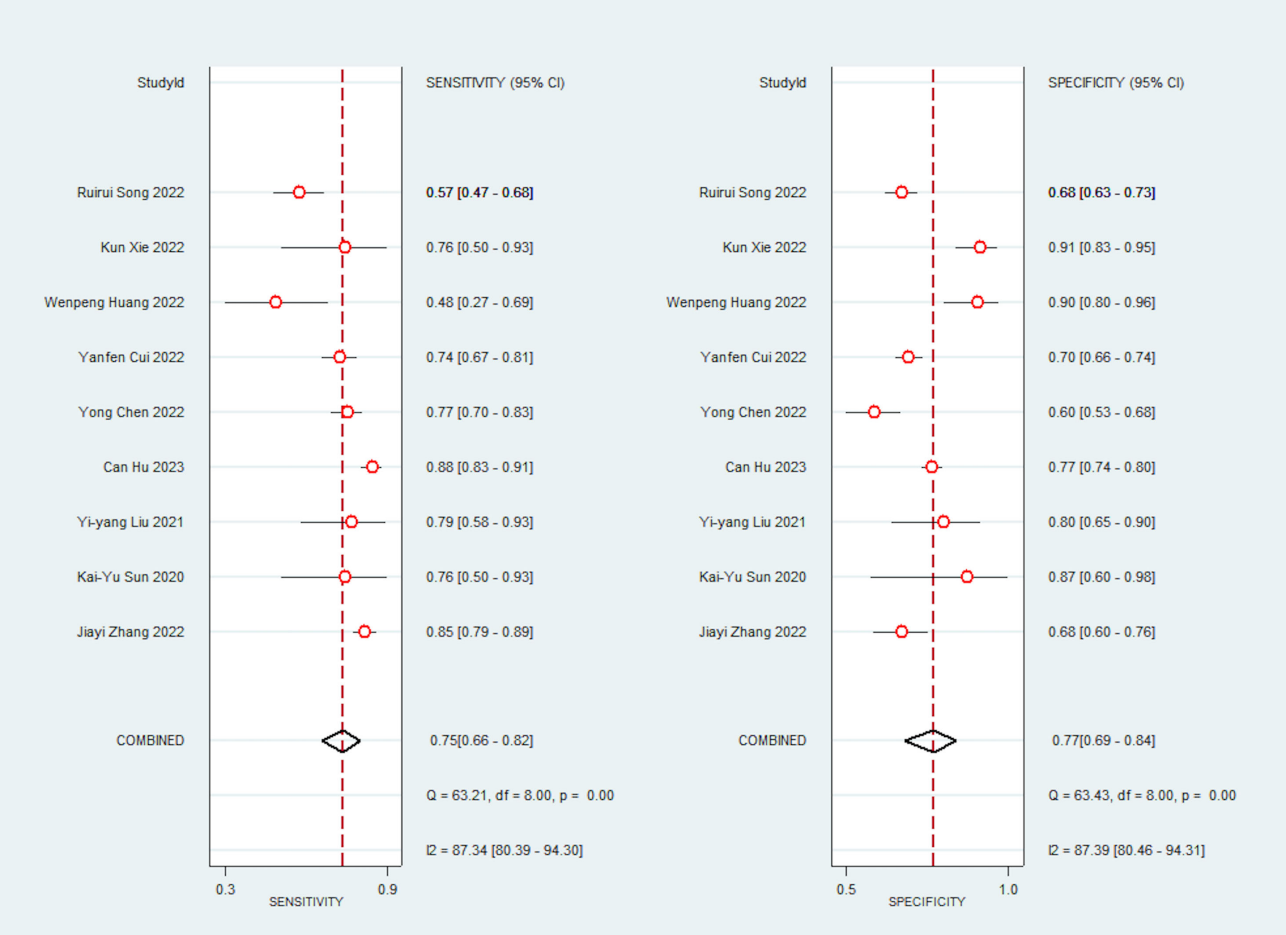
Forest plots of SEN and SPE with corresponding 95% CIs of AI.

Due to significant heterogeneity in the pooled analyses, a random-effects coefficient binary regression model was utilized. Among the 9 studies that reported AI diagnostic accuracy (I^2 ^= 87.37%), when comparing to the gold standard, the pooled weighted values were as follows: SEN 0.75 (95% CI: 0.66, 0.82), SPE 0.77 (95% CI: 0.69, 0.84), PLR 3.30 (95% CI: 2.40, 4.50), NLR 0.32 (95% CI: 0.23, 0.44), DOR 10.00 (95% CI: 6.00, 17.00), and the areas under the ROC curve [Area Under Curve (AUC)] 0.83 (95% CI: 0.79, 0.86). The forest plot can be seen in **Figure 3**. In sensitivity analyses, the results analyzed by fixed effects model were inconsistent with those by random effects model, which indicated that results were not robust.

**Figure 4:**
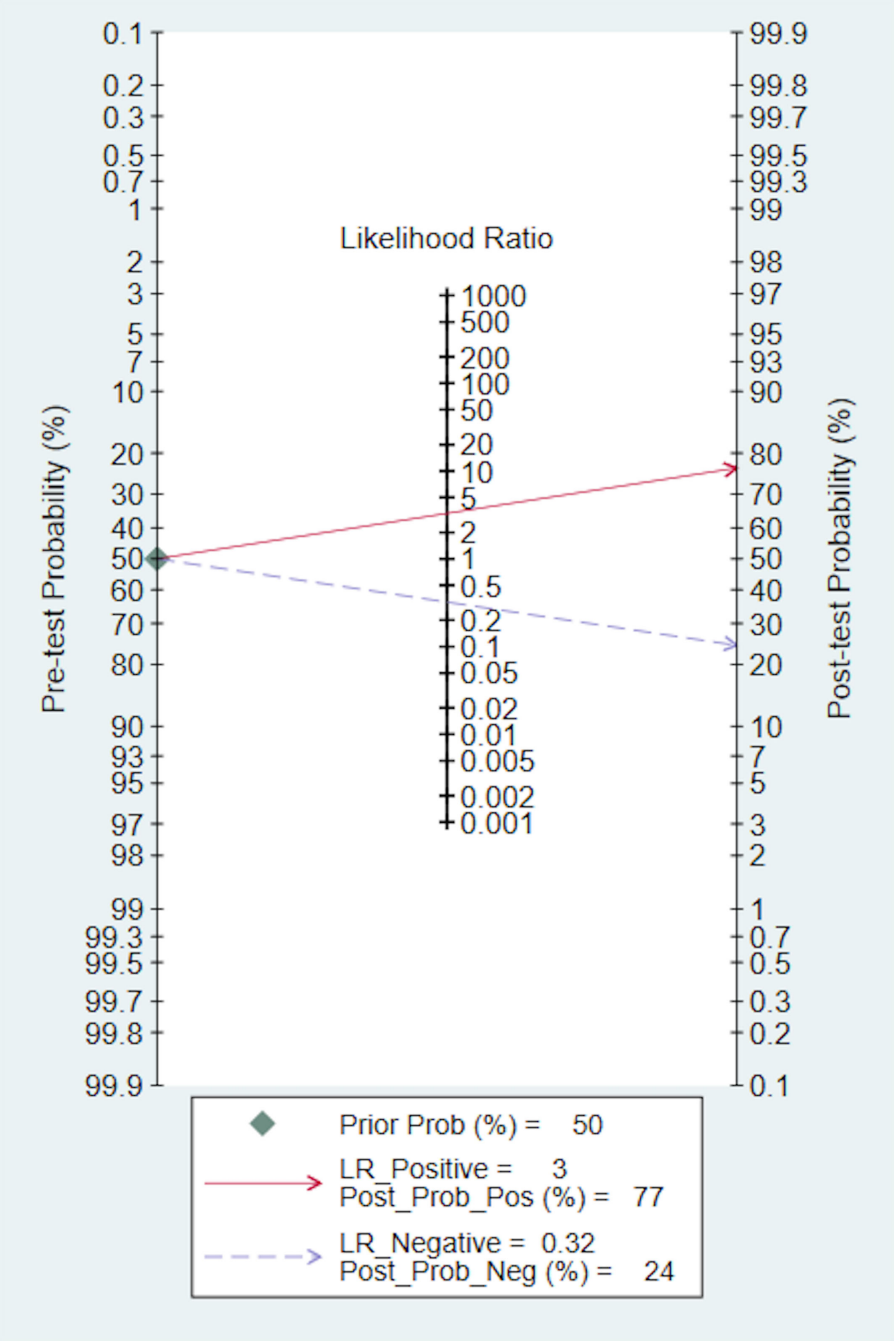
Fagan normogram of AI for the predicting response to NACT.

We also conducted a Fagan nomogram to explore the clinical application of AI. Assuming a 50% response rate to NACT in GC patients, the Fagan nomogram indicates a posteriori probability of a response rate of 77% if the test is positive and 24% if the test is negative, as shown in **Figure 4**.

Additionally, we performed subgroup analysis based on pre-designed factors with a random-effects coefficient binary regression model. In terms of the cut-off value factor, the ROC analysis subgroup had a lower pooled SPE than the not reported subgroup (0.69 [95% CI: 0.57, 0.81] vs. 0.81 [95% CI: 0.74, 0.88], P=0.00), but there was no significant difference in SEN (0.74 [95% CI: 0.61, 0.87] vs. 0.76 [95% CI: 0.66, 0.86], P=0.11). Regarding the approach of predicting response factor, the CT-based radiomics subgroup was more specific than the CT-based deep learning subgroup (0.80 [95% CI: 0.72, 0.88] vs. 0.72 [95% CI: 0.60, 0.84], P=0.02), but had equivalent SEN (0.69 [95% CI: 0.60, 0.78] vs. 0.83 [95% CI: 0.76, 0.90], P=0.40). Lastly, concerning the gold standard factor, the NCCN guideline subgroup was inferior to the other subgroups, with a lower pooled SPE (0.76 [95% CI: 0.66, 0.85] vs. 0.79 [95% CI: 0.68, 0.90], P=0.04), but was comparable in SEN (0.78 [95% CI: 0.69, 0.87] vs. 0.71 [95% CI: 0.58, 0.85], P=0.37). The results of the subgroup analysis are presented in **Table 2**. These findings suggest that the cut-off value, approach of predicting response, and gold standard may be potential sources of heterogeneity. In sensitivity analyses, the results analyzed by fixed effects model were consistent with those by random effects model, which indicated that results were robust.

**Table 2:** Subgroup analysis of AI for predicting response to NACT.

Factor	Subgroups	No.	pSEN [95%CI]	P value	pSPE [95%CI]	P value
Cut-off value	ROC analysis	3	0.74 [0.61 - 0.87]	P = 0.11	0.69 [0.57 - 0.81]	P = 0.00*
	NR	6	0.76 [0.66 - 0.86]		0.81 [0.74 - 0.88]	
Location of cancer	esophagogastric junction cancer	2	0.75 [0.59 - 0.92]	P = 0.31	0.84 [0.73 - 0.94]	P = 0.49
	gastric cancer	7	0.75 [0.66 - 0.84]		0.74 [0.66 - 0.82]	
Cohorts (No.)	one	2	0.79 [0.60 - 0.97]	P = 0.62	0.83 [0.68 - 0.98]	P = 0.69
	more than one	7	0.75 [0.66 - 0.83]		0.76 [0.68 - 0.84]	
Approach of predicting response	CT-based deep learning	3	0.83 [0.76 - 0.90]	P = 0.40	0.72 [0.60 - 0.84]	P = 0.02*
	CT-based radiomics	6	0.69 [0.60 - 0.78]		0.80 [0.72 - 0.88]	
Gold standard	NCCN guideline	5	0.78 [0.69 - 0.87]	P = 0.37	0.76 [0.66 - 0.85]	P = 0.04*
	Other guidelines	4	0.71 [0.58 - 0.85]		0.79 [0.68 - 0.90]	

AI, artificial intelligence; NACT, Neoadjuvant chemotherapy; pSEN, pooled sensitivity; pSPE, pooled specificity; ROC, Receiver Operating Characteristic; NR, not reported; CT, computed tomography; NCCN, National Comprehensive Cancer Network; *indicated statistical significance.

### 3.4 Comparison of Diagnostic Test Accuracy of AI, CM and IM

**Figure 5:**
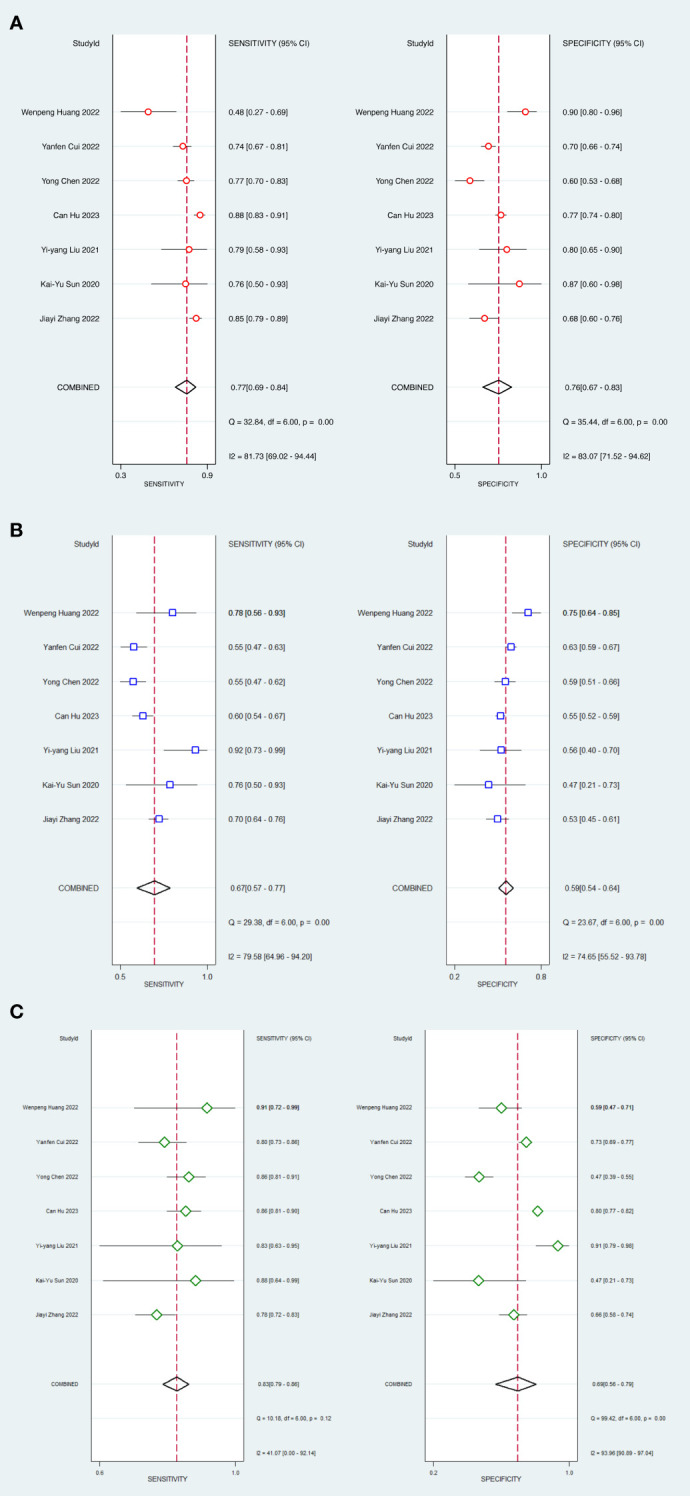
Forest plots of SEN and SPE with corresponding 95% CIs of AI, CM and IM. **(A)** AI; **(B)** Clinical model; **(C)** Integrated model.

**Figure 6:**
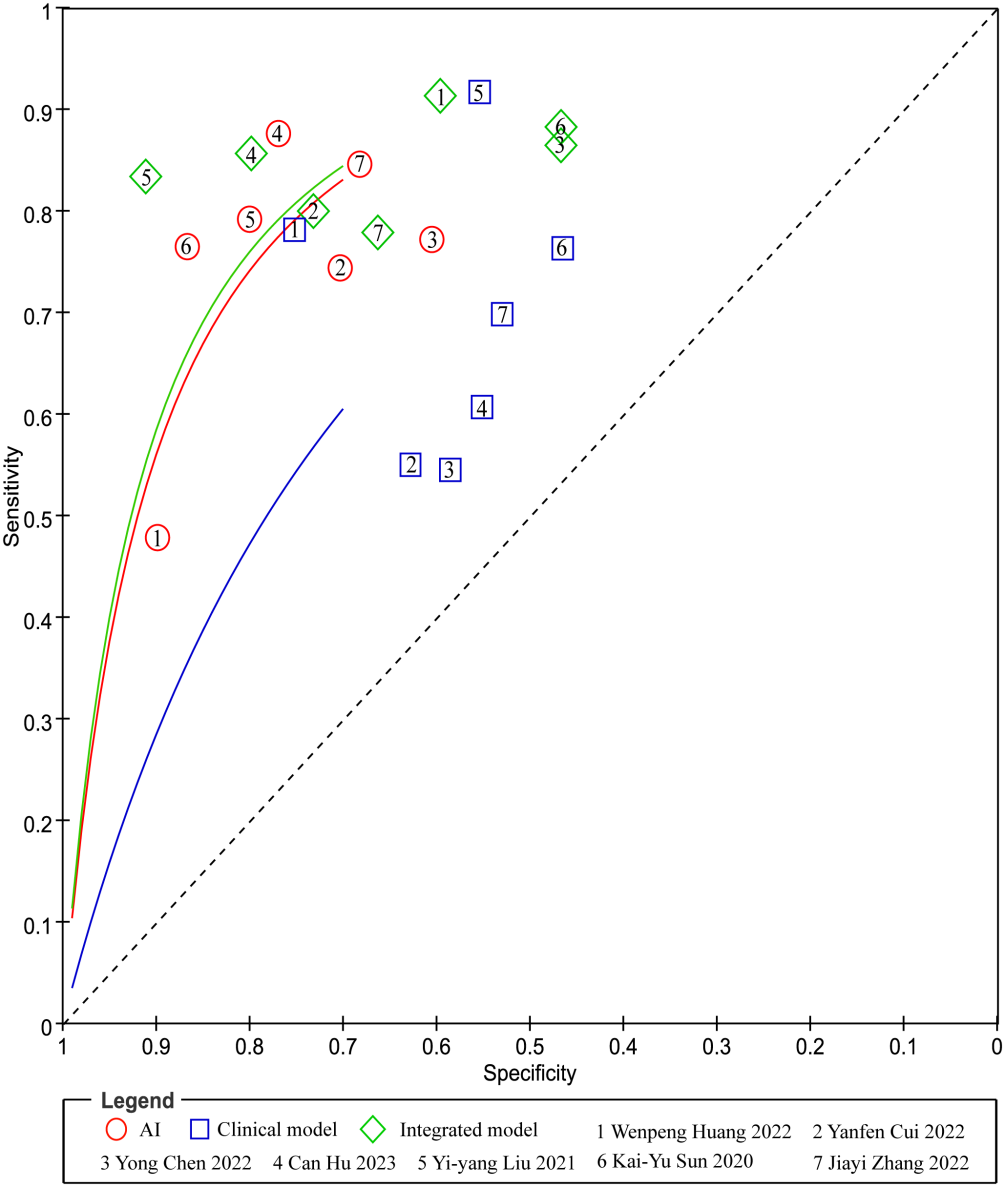
Pairs of observed values of sensitivity and specificity for AI, CM and IM to predict response.

In head-to-head comparison studies, a random-effects coefficient binary regression model was employed to account for significant heterogeneity. The pooled weighted values for AI were as follows: SEN 0.77 (95% CI: 0.69, 0.84), SPE 0.79 (95% CI: 0.70, 0.85), PLR 3.60 (95% CI: 2.60, 5.10), NLR 0.29 (95% CI: 0.22, 0.39), DOR 12.00 (95% CI: 8.00, 20.00), and AUC 0.85 (95% CI: 0.81, 0.87). For CM, the pooled weighted values were as follows: SEN 0.67 (95% CI: 0.57, 0.77), SPE 0.59 (95% CI: 0.54, 0.64), PLR 1.60 (95% CI: 1.30, 2.00), NLR 0.55 (95% CI: 0.40, 0.77), DOR 3.00 (95% CI: 2.00, 5.00), and AUC 0.64 (95% CI: 0.60, 0.68). Lastly, for IM, the pooled weighted values were as follows: SEN 0.83 (95% CI: 0.79, 0.86), SPE 0.69 (95% CI: 0.56, 0.79), PLR 2.60 (95% CI: 1.80, 3.80), NLR 0.24 (95% CI: 0.19, 0.31), DOR 11.00 (95% CI: 6.00, 19.00), and AUC 0.85 (95% CI: 0.82, 0.88). In sensitivity analyses, the results analyzed by fixed effects model were inconsistent with those by random effects model, which indicated that results were not robust. The forest plots and HSROC curves can be seen in **Figure 5** and **Figure 6**, respectively. **Table 3** presents the results of a pairwise comparison between the three models, revealing no significant difference in diagnostic accuracy, except for a statistical difference in SEN between AI and IM.

**Table 3:** Pairwise comparison of three models for pSEN and pSPE.

Model	No.	pSEN [95%CI]	P value	pSPE [95%CI]	P value
AI vs. Clinical model	7	0.78 [0.70 - 0.85] vs. 0.67 [0.58 - 0.77]	P = 0.30	0.75 [0.69 - 0.81] vs. 0.59 [0.51 - 0.66]	P = 0.64
Integrated vs. Clinical model	7	0.84 [0.79 - 0.88] vs. 0.65 [0.58 - 0.73]	P = 0.28	0.68 [0.60 - 0.77] vs. 0.59 [0.49 - 0.68]	P = 0.63
AI vs. Integrated model	7	0.78 [0.72 - 0.84] vs. 0.84 [0.79 - 0.89]	P = 0.00*	0.76 [0.68 - 0.85] vs. 0.68 [0.58 - 0.79]	P = 0.28

pSEN, pooled sensitivity; pSPE, pooled specificity; AI, artificial intelligence; *indicated statistical significance.

In the ROC-analysis cut-off subgroup, AI outperformed IM in assessing the response of GC to NACT, with a notably higher pooled SEN (0.80 [95% CI: 0.71, 0.87] vs. 0.79 [95% CI: 0.72, 0.85], P=0.00), but a lower pooled SPE (0.70 [95% CI: 0.65, 0.74] vs. 0.72 [95% CI: 0.67, 0.76], P=0.00). Conversely, in the not reported cut-off subgroup, IM performed better than AI in assessing the response of GC to NACT, with a higher pooled SEN (0.87 [95% CI: 0.81, 0.92] vs. 0.77 [95% CI: 0.69, 0.85], P=0.00) and a similar pooled SPE (0.68 [95% CI: 0.53, 0.83] vs. 0.80 [95% CI: 0.68, 0.91], P=0.91). The variations in the statistical significance of SPE suggest that the cut-off value may be a source of heterogeneity. In the two subgroups relating to the location of cancer, IM was more effective than AI in predicting the response of GC to NACT, with a higher pooled SEN (esophagogastric junction cancer: 0.88 [95% CI: 0.80, 0.97] vs. 0.79 [95% CI: 0.66, 0.91], P=0.03; GC: 0.81 [95% CI: 0.77, 0.86] vs. 0.78 [95% CI: 0.74, 0.83], P=0.00). These findings indicate that the location of the cancer was not the cause of heterogeneity. In more than one cohort subgroup, IM preceded AI in predicting the response of GC to NACT, with a higher SEN (0.84 [95% CI: 0.78, 0.90] vs. 0.78 [95% CI: 0.71, 0.85], P=0.00), but a comparable SEN in the one-cohort subgroup (0.86 [95% CI: 0.75, 0.97] vs. 0.78 [95% CI: 0.65, 0.91], P=0.09). The variations in the significance of SEN suggest that the number of cohorts might be a source of heterogeneity. In the training cohort subgroup, IM outperformed AI in assessing the response of GC to NACT, with a higher SEN (0.88 [95% CI: 0.82, 0.94] vs. 0.80 [95% CI: 0.72, 0.88], P=0.00), but a lower SPE (0.77 [95% CI: 0.69, 0.85] vs. 0.81 [95% CI: 0.74, 0.88], P=0.03). Similarly, in the internal validation cohort subgroup, IM was more effective than AI in assessing the response of GC to NACT, with a higher SEN (0.84 [95% CI: 0.78, 0.89] vs. 0.80 [95% CI: 0.74, 0.86], P=0.00), but a lower SPE (0.73 [95% CI: 0.68, 0.79] vs. 0.76 [95% CI: 0.70, 0.81], P=0.00). However, in the external validation cohort subgroup, IM and AI demonstrated similar performance in assessing the response of GC to NACT, indicating that diagnostic accuracy varied depending on the cohort type. In the CT-based deep learning subgroup, AI proved to be superior to IM in assessing the response of GC to NACT, with a higher SEN (0.83 [95% CI: 0.77, 0.88] vs. 0.81 [95% CI: 0.76, 0.87], P=0.00), but a lower SPE (0.72 [95% CI: 0.68, 0.76] vs. 0.74 [95% CI: 0.70, 0.78], P=0.00). Conversely, in the CT-based radiomics subgroup, IM outperformed AI in SEN (0.85 [95% CI: 0.80, 0.1] vs. 0.71 [95% CI: 0.62, 0.80], P=0.00), but showed similar SPE (0.63 [95% CI: 0.44, 0.83] vs. 0.80 [95% CI: 0.67, 0.94], P=0.62). In the gold standard factor analysis, the performance of the NCCN guideline subgroup mirrored that of CT-based DL, but the details are not provided. The variations in the significance of SEN suggest that the approach to predicting response and different gold standards may be sources of heterogeneity. The results of the subgroup analysis can be found in **Table 4**. In sensitivity analyses, the results analyzed by fixed effects model were consistent with those by random effects model, which indicated that results were robust.

**Table 4:** Pairwise comparison of three models for pSEN and pSPE in subgroup analysis.

Factors	Subgroups	Model	No.	pSEN [95%CI]	P value	pSPE [95%CI]	P value
Cut-off value	ROC analysis	AI VS CM	2	0.80 [0.73-0.87] vs. 0.63 [0.53-0.73]	P = 0.82	0.69 [0.64-0.74] vs. 0.59 [0.53-0.65]	P = 0.17
		IM VS CM	2	0.79 [0.73 - 0.85] vs. 0.63 [0.55 - 0.71]	P = 0.41	0.71 [0.66 - 0.77] vs. 0.59 [0.52 - 0.65]	P = 0.32
		AI VS IM	2	0.80 [0.71 - 0.87] vs. 0.79 [0.72 - 0.85]	P = 0.00*	0.70 [0.65 - 0.74] vs. 0.72 [0.67 - 0.76]	P = 0.00*
	NR	AI VS CM	5	0.76 [0.65 - 0.88] vs. 0.71 [0.58 - 0.84]	P = 0.43	0.78 (0.70 - 0.86) vs. 0.59 (0.48 - 0.70)	P = 0.86
		IM VS CM	5	0.87 [0.81 - 0.92] vs. 0.68 [0.56 - 0.79]	P = 0.83	0.68 [0.55 - 0.81] vs. 0.59 [0.45 - 0.73]	P = 0.89
		AI VS IM	5	0.77 [0.69 - 0.85] vs. 0.87 [0.81 - 0.92]	P = 0.00*	0.80 [0.68 - 0.91] vs. 0.68 [0.53 - 0.83]	P = 0.91
Location of cancer	esophagogastric junction cancer	AI VS CM	2	0.74 [0.53 - 0.95] vs. 0.67 [0.43 - 0.92]	P = 0.89	0.84 [0.74 - 0.93] vs. 0.64 [0.49 - 0.79]	P = 0.63
		IM VS CM	2	0.85 [0.79 - 0.90] vs. 0.65 [0.56 - 0.74]	P = 0.71	0.74 [0.65 - 0.82] vs. 0.64 [0.53 - 0.74]	P = 0.47
		AI VS IM	2	0.79 [0.66 - 0.91] vs. 0.88 [0.80 - 0.97]	P = 0.03*	0.81 [0.73 - 0.89] vs. 0.73 [0.64 - 0.82]	P = 0.14
	gastric cancer	AI VS CM	5	0.78 [0.72 - 0.85] vs. 0.66 [0.57 - 0.75]	P = 0.29	0.69 [0.64 - 0.73] vs. 0.57 [0.52 - 0.63]	P = 0.23
		IM VS CM	5	0.81 [0.76 - 0.87] vs. 0.65 [0.56 - 0.74]	P = 0.44	0.66 [0.56 - 0.76] vs. 0.55 [0.44 - 0.66]	P = 0.97
		AI VS IM	5	0.78 [0.74 - 0.83] vs. 0.81 [0.77 - 0.86]	P = 0.00*	0.72 [0.61 - 0.83] vs. 0.67 [0.55 - 0.78]	P = 0.36
Cohorts (No.)	one	AI VS CM	2	0.78 [0.65 - 0.91] vs. 0.85 [0.75 - 0.96]	P = 0.08	0.82 [0.72 - 0.91] vs. 0.53 0.41 - 0.66]	P = 0.24
		IM VS CM	2	0.86 [0.75 - 0.97] vs. 0.85 [0.75 - 0.96]	P = 0.32	0.76 [0.54 - 0.98] vs. 0.52 [0.24 - 0.80]	P = 0.29
		AI VS IM	2	0.78 [0.65 - 0.91] vs. 0.86 [0.75 - 0.97]	P = 0.09	0.84 [0.66 - 1.00] vs. 0.76 [0.53 - 0.99]	P = 0.69
	more than 1	AI VS CM	5	0.78 [0.71 - 0.85] vs. 0.62 [0.52 - 0.72]	P = 0.73	0.73 [0.67 - 0.80] vs. 0.60 [0.52 - 0.69]	P = 0.52
		IM VS CM	5	0.83 [0.79 - 0.87] vs. 0.61 [0.55 - 0.67]	P = 0.41	0.67 [0.58 - 0.75] vs. 0.61 [0.51 - 0.70]	P = 0.38
		AI VS IM	5	0.78 [0.71 - 0.85] vs. 0.84 [0.78 - 0.90]	P = 0.00*	0.74 [0.65 - 0.83] vs. 0.66 [0.56 - 0.77]	P = 0.34
Type of cohorts	T	AI VS CM	5	0.79 (0.69-0.90) VS 0.68 (0.55-0.82)	P = 0.73	0.81 (0.74-0.87) vs.0.63 (0.54-0.72)	P = 0.68
		IM VS CM	5	0.88 [0.84 - 0.92] 0.63 [0.57 - 0.69]	P = 0.67	0.77 [0.69 - 0.84] vs.0.64 [0.54 - 0.73]	P = 0.52
		AI VS IM	5	0.80 [0.72 - 0.88] vs. 0.88 [0.82 - 0.94]	P = 0.00*	0.81 [0.74 - 0.88] vs. 0.77 [0.69 - 0.85]	P = 0.03*
	I	AI VS CM	6	0.81 [0.71 - 0.90] vs. 0.63 [0.50 - 0.75]	P = 0.85	0.76 [0.71 - 0.81] vs. 0.64 [0.58 - 0.70]	P = 0.07
		IM VS CM	6	0.84 [0.76 - 0.92] vs. 0.63 [0.50 - 0.76]	P = 0.65	0.73 [0.67 - 0.79] vs. 0.64 [0.58 - 0.70]	P = 0.06
		AI VS IM	6	0.80 [0.74 - 0.86] vs. 0.84 [0.78 - 0.89]	P = 0.00*	0.76 [0.70 - 0.81] vs. 0.73 [0.68 - 0.79]	P = 0.00*
	E	AI VS CM	7	0.80 [0.70-0.91] vs. 0.61 [0.46-0.77]	P = 0.65	0.65 [0.54-0.76] vs. 0.58 [0.46-0.70]	P = 0.75
		IM VS CM	7	0.84 [0.74 - 0.93] vs. 0.60 [0.45 - 0.76]	P = 0.44	0.61 [0.47 - 0.76] vs. 0.58 [0.43 - 0.73]	P = 0.77
		AI VS IM	7	0.80 [0.69 - 0.92] vs. 0.84 [0.74 - 0.94]	P = 0.09	0.65 [0.52 - 0.79] vs. 0.61 [0.47 - 0.76]	P = 0.68
Approach of predicting response	CT-based deep learning	AI VS CM	3	0.83 [0.78 - 0.88] vs. 0.62 [0.54 - 0.70]	P = 0.74	0.73 [0.69 - 0.77] vs. 0.58 [0.53 - 0.63]	P = 0.29
		IM VS CM	3	0.81 [0.77 - 0.86] vs. 0.62 [0.56 - 0.69]	P = 0.34	0.74 [0.69 - 0.79] vs. 0.58 [0.51 - 0.64]	P = 0.56
		AI VS IM	3	0.83 [0.77 - 0.88] vs. 0.81 [0.76 - 0.87]	P = 0.00*	0.72 [0.68 - 0.76] vs. 0.74 [0.70 - 0.78]	P = 0.00*
	CT-based radiomics	AI VS CM	4	0.71 [0.57 - 0.85] vs. 0.74 [0.60 - 0.88]	P = 0.25	0.79 [0.68 - 0.90] vs. 0.60 [0.46 - 0.75]	P = 0.71
		IM VS CM	4	0.87 [0.80 - 0.95] vs. 0.73 [0.60 - 0.87]	P = 0.91	0.63 [0.47 - 0.80] vs. 0.61 [0.44 - 0.77]	P = 0.76
		AI VS IM	4	0.71 [0.62 - 0.80] vs. 0.85 [0.80 - 0.91]	P = 0.00*	0.80 [0.67 - 0.94] vs. 0.63 [0.44 - 0.83]	P = 0.62
Gold standard	NCCN guideline	AI VS CM	3	0.83 [0.78 - 0.88] vs. 0.62 [0.54 - 0.70]	P = 0.74	0.73 [0.69 - 0.77] vs. 0.58 [0.53 - 0.63]	P = 0.29
		IM VS CM	3	0.81 [0.77 - 0.86] vs. 0.62 [0.56 - 0.69]	P = 0.34	0.74 [0.69 - 0.79] vs. 0.58 [0.51 - 0.64]	P = 0.56
		AI VS IM	3	0.83 [0.77 - 0.88] vs. 0.81 [0.76 - 0.87]	P = 0.00*	0.72 [0.68 - 0.76] vs. 0.74 [0.70 - 0.78]	P = 0.00*
	Other guidelines	AI VS CM	4	0.71 [0.57 - 0.85] vs. 0.74 [0.60 - 0.88]	P = 0.25	0.79 [0.68 - 0.90] vs. 0.60 [0.46 - 0.75]	P = 0.71
		IM VS CM	4	0.87 [0.80 - 0.95] vs. 0.73 [0.60 - 0.87]	P = 0.91	0.63 [0.47 - 0.80] vs. 0.61 [0.44 - 0.77]	P = 0.76
		AI VS IM	4	0.71 [0.62 - 0.80] vs. 0.85 [0.80 - 0.91]	P = 0.00*	0.80 [0.67 - 0.94] vs. 0.63 [0.44 - 0.83]	P = 0.62

pSEN, pooled sensitivity; pSPE, pooled specificity; AI, artificial intelligence; CM, clinical model; IM, integrated model; ROC, Receiver Operating Characteristic; NR, not reported; T, training cohort; I, internal validation cohort; E, external validation cohort; CT, computed tomography; NCCN, National Comprehensive Cancer Network; *indicated statistical significance.

### 3.5 Comparison among cohorts in three models

We conducted pairwise comparisons of three cohorts on the same test with a random-effects coefficient binary regression model to determine if there were differences in performance across the cohorts, as depicted in **Table 5**. In sensitivity analyses, the results analyzed by fixed effects model were inconsistent with those by random effects model, which indicated that results were not robust.

**Table 5:** Pairwise comparison of three cohorts for pSEN and pSPE.

	AI		Clinical model		Integrated model	
	pSEN [95%CI]	pSPE [95%CI]	pSEN [95%CI]	pSPE [95%CI]	pSEN [95%CI]	pSPE [95%CI]
T VS I	0.80 [0.71 - 0.88]	0.80 [0.74 - 0.85]	0.68 [0.55 - 0.82]	0.62 [0.55 - 0.70]	0.88 [0.84 - 0.91]	0.77 [0.70 - 0.84]
	0.81 [0.72 - 0.90]	0.76 [0.70 - 0.83]	0.63 [0.48 - 0.78]	0.64 [0.56 - 0.72]	0.84 [0.78 - 0.89]	0.71 [0.61 - 0.80]
	P = 0.03*	P = 0.00*	P = 0.73	P = 0.07	P = 0.00*	P = 0.10
T VS E	0.79 (0.66-0.92)	0.81 (0.74-0.89)	0.67 (0.53-0.82)	0.63 (0.51-0.75)	0.88 [0.83 - 0.94]	0.77 [0.65 - 0.90]
	0.80 (0.70-0.91)	0.65 (0.56-0.75)	0.62 (0.49-0.75)	0.58 (0.48-0.69)	0.81 [0.74 - 0.88]	0.61 [0.47 - 0.76]
	P = 0.17	P = 0.73	P = 0.71	P = 0.67	P = 0.04*	P = 0.77
I VS E	0.80 [0.70 - 0.91]	0.77 [0.69 - 0.85]	0.63 [0.46 - 0.80]	0.63 [0.52 - 0.75]	0.84 [0.76 - 0.93]	0.70 [0.54 - 0.85]
	0.81 [0.72 - 0.90]	0.65 [0.57 - 0.73]	0.62 [0.47 - 0.76]	0.58 [0.48 - 0.68]	0.82 [0.74 - 0.89]	0.61 [0.47 - 0.75]
	P = 0.09	P = 0.39	P = 0.67	P = 0.69	P = 0.06	P = 0.90

pSEN, pooled sensitivity; pSPE, pooled specificity; AI, artificial intelligence; T, training cohort; I, internal validation cohort; E, external validation cohort; *indicated statistical significance.

In the AI model, the accuracy differed between the training cohort and the internal validation cohort. The pooled SEN was lower in the training cohort (0.80 [95% CI: 0.71, 0.88]) compared to the internal validation cohort (0.81 [95% CI: 0.72, 0.90]), with a statistically significant P-value of 0.03. However, the pooled SPE was higher in the training cohort (0.80 [95% CI: 0.74, 0.85]) compared to the internal validation cohort (0.76 [95% CI: 0.70, 0.83]), with a statistically significant P-value of 0.00.

In the IM model, the training cohort exhibited superior performance in terms of pooled SEN (0.88 [95% CI: 0.84, 0.91]) compared to the internal validation cohort (0.84 [95% CI: 0.78, 0.89]), with a statistically significant P-value of 0.00. However, they demonstrated similar performance in terms of pooled SPE (0.77 [95% CI: 0.70, 0.84] vs. 0.71 [95% CI: 0.61, 0.80]), with a non-significant P-value of 0.10. Additionally, the training cohort displayed higher pooled SEN (0.88 [95% CI: 0.83, 0.94]) compared to the external validation cohort (0.81 [95% CI: 0.74, 0.88]), with a statistically significant P-value of 0.04. They exhibited similar performance in terms of pooled SPE (0.77 [95% CI: 0.65, 0.90] vs. 0.61 [95% CI: 0.47, 0.76]), with a non-significant P-value of 0.77. In sensitivity analyses, the results analyzed by fixed effects model were consistent with those by random effects model, which indicated that results were robust.

### 3.6 Risk-of-bias assessment

**Figure 7:**
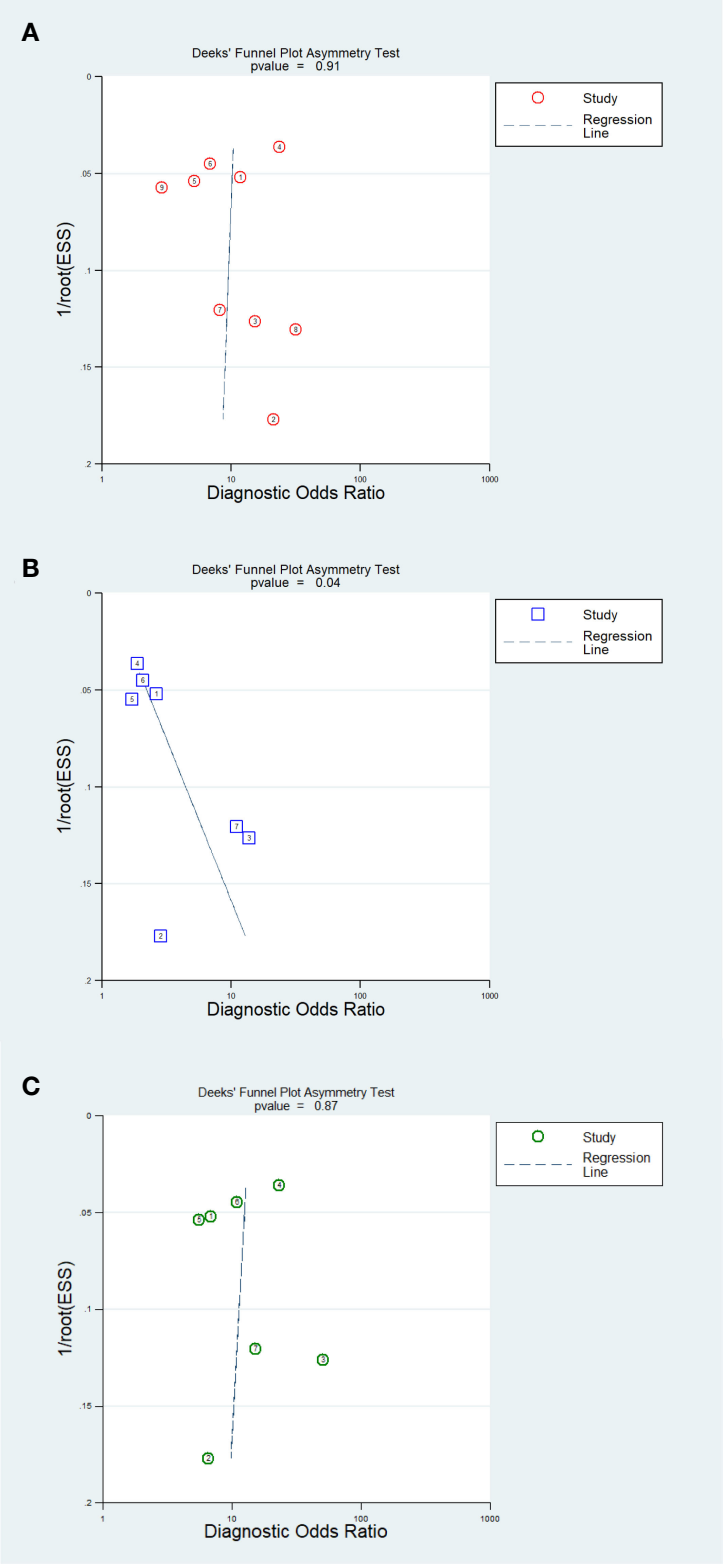
Funnel plot of publication bias. **(A)** AI; **(B)** Clinical model; **(C)** Integrated model.

The results of the Deeks’ funnel plot asymmetry test demonstrated that no significant evidence of publication bias was observed in the analysis of the AI model (P=0.91) and IM analysis (P=0.87). However, the CM analysis revealed compelling evidence of publication bias (P=0.04). The Deeks’ funnel plot, which showcases these findings, is presented in **Figure 7**.

## 4 Discussion

### 4.1 Principal findings

To the best of our knowledge, this review represents the first attempt to comprehensively summarize the diagnostic accuracy of CT-based deep learning or radiomics in predicting the response of GC to NACT. This study consists of two main parts: first, we evaluated the diagnostic accuracy of AI models; and second, we compared the accuracy of AI, CM, and IM models in head-to-head studies. The review showed that AI is an effective tool for predicting the response of GC patients to NACT. It has been observed that when AI is combined with clinical features, it becomes more sensitive than the AI model alone. However, in the ROC analysis subgroup, the CT-based DL subgroup, and the NCCN guideline subgroup, the AI model was more sensitive than IM. Subjects can be categorized according to heterogeneous factors, with a higher degree of homogeneity across subjects within the group. Therefore, the results of subgroup analysis are more reliable. In conclusion, AI is most sensitive for predicting the response of GC patients to NACT when assessing tumor grade with reference to the NCCN guidelines, extracting tumor characteristics using a CT-based DL approach, and determining the cut-off value of the test using ROC curve.

The threshold effect, resulting from the use of different diagnostic cut-off values in various studies, has led to inconsistent findings with high SEN and low SPE, and vice versa (38). To address this, we conducted a subgroup analysis based on cut-off values reported in the literature. We found a statistically significant difference in SPE between two subgroups when analyzing AI studies using different reporting methods for cut-off values (ROC analysis: 0.69 vs. not reported: 0.81). Furthermore, the conclusions for SEN were opposite in head-to-head comparisons, and the statistical significance of SPE differed when comparing AI and IM. These findings confirm our hypothesis about the threshold effect and suggest that the heterogeneity observed in subgroup analysis may be attributed to variations in cut-off values.

It has been reported that NACT improves 5-year overall survival and progression-free survival in patients with esophagogastric junction cancer (39). However, it is worth noting that this study included two articles on esophagogastric junction cancer, which may have affected the reliability of the results due to the different types of diseases analyzed. Interestingly, our analysis showed no difference between subgroups in AI studies when considering tumor location. Additionally, the diagnostic accuracy of pairwise comparisons did not change significantly before and after subgroup analysis, indicating that tumor location did not contribute to heterogeneity or affect the robustness of the results.

To assess the exact performance of AI algorithms, it is recommended to conduct external validation using independent datasets (40). Consequently, the majority of the included studies (5 out of 7) performed external validation to ensure the authenticity and generalizability of their findings. Our hypothesis considered that variations in test intervals and populations might result in different test performances across various cohorts. We categorized the cohorts into training cohorts, internal validation cohorts, and external validation cohorts based on their number and type. No significant difference was observed between subgroups in AI analysis when considering the number of cohorts. However, in head-to-head comparisons, the diagnostic accuracy of AI and IM showed inconsistency between the two subgroups. Depending on the cohort type, AI performed better in internal validation cohorts in terms of sensitivity but exhibited lower specificity compared to the training cohorts in predicting the response to NACT. Moreover, in head-to-head comparisons, IM outperformed AI with higher sensitivity but lower specificity in both training and internal validation cohorts. These findings highlight the influence of the number and type of cohorts on the results and emphasize the importance of exploring heterogeneity.

Both DL and radiomics are rapidly advancing and promising approaches that can predict patient outcomes after diagnosis and treatment (41–44). However, in radiomics, manual delineation of tumors is required, whereas in DL, no human involvement is necessary. Our analysis showed that in the AI model, the subgroup using CT-based radiomics exhibited higher pooled SPE compared to the subgroup using CT-based DL. In head-to-head comparisons, the conclusions for SEN were opposite when comparing AI with IM in the two subgroups. That is to say, in the CT-based DL subgroup, IM performed better than AI with higher specificity.

The NCCN guideline are widely accepted and referenced for evaluating tumor regression grade (45). However, some studies have used other criteria to assess tumor regression grade (46–48). In certain subgroups, other guidelines have shown higher pooled SPE than the NCCN guidelines when using AI models. In head-to-head comparisons, the SEN conclusions were contradictory when comparing AI with IM in two subgroups. Additionally, in the NCCN guideline subgroup, IM exhibited superiority over AI with higher specificity. The variability observed in predicting response and the selection of guidelines may contribute to the identified heterogeneity.

### 4.2 Practical implications

Preoperative evaluation of GC patients scheduled for NACT is conducive to clinical decision making. The prognosis of GC patients is poor and missed diagnosis of patients may bring serious consequences. Moreover, NACT is effective in the treatment of GC patients. Therefore, in practice, we should choose a method of higher SEN, as much as possible to find suspicious patients. In conclusion, it is best to construct CT-based DL model of AI rather than CT-based radiomics to predict the response of GC patients to NACT because of a higher SEN. When evaluating GC regression grade, the NCCN guideline should be referenced, because of a higher SEN, and in the NCCN subgroup, AI model was more sensitive than IM and CM. By analyzing preoperative CT images of patients, AI can avoid the harm caused by pathological examination and reduce the medical burden. AI has demonstrated a level above that of clinicians and imaging physicians. In later practice, AI can be used more widely as an assistant tool for clinicians. If we can conduct rigorously designed diagnostic accuracy studies and head-to-head comparative studies, the conclusions of SEN and SPE will be more accurate.

In policy, governments should consider investments not only in acquiring computer equipment and providing personnel training for hospitals but also in supporting scientific research that enhances the accuracy of these diagnostic tests. Future research endeavors should prioritize updating AI technology and augmenting its intelligence to achieve even greater precision in predicting patients’ responses to NACT. It is recommended that the threshold determination method be indicated in the publication when studying the diagnostic accuracy of a test. In the process of validation, external validation cohort using independent datasets is recommended.

### 4.3 Limitations

There were several limitations in the review. Firstly, the limited number of included studies impacted the reliability and generalizability of the results. Specifically, there was a lack of head-to-head comparative studies. Secondly, the overfitting of the AI algorithm could lead to an excessive adaptation to the training dataset, hindering accurate predictions for new datasets (40). Therefore, it is crucial to ensure that the selected target population is representative. In this review, there were three times as many male participants as female participants, and all the articles were sourced from Asia. This skewed representation may result in a matching model that is more suitable for a specific population cohort, introducing bias. Thirdly, variations in the baselines of the articles, such as cut-off values, number and type of cohorts, and gold standard, among others, necessitated numerous subgroup analyses to evaluate their impact on the stability of the conclusions. Fourthly, none of the included articles reported the cut-off value, and most did not provide an explanation for how the cut-off value was determined. This lack of information affects the reliability of the diagnostic conclusions. Fifthly, in selection and data extraction phases, although the reviewers were trained beforehand and a third reviewer was involved in the discussions, bias was inevitable due to staff subjectivity. We included only English language literature, which was also a source of bias. Reporting bias should be checked if a study has multiple outcome indicators but only reports statistically significant results, but this was not the case in this study. Additionally, factors that could potentially affect the accuracy estimates, such as the clinical stage of cancer, type of AI, and NACT regimen, were not thoroughly explored due to insufficiently detailed data or the lack of a basis for grouping.

## 5 Conclusion

AI is a highly effective tool for accurately predicting the response of GC patients to NACT. Furthermore, CT-based DL model in AI is sensitive to extract tumor features and predict the response. It is critical to conduct rigorously designed, high-quality diagnostic accuracy studies to validate the conclusions.

## Data availability statement

The original contributions presented in the study are included in the article/**Supplementary Material**. Further inquiries can be directed to the corresponding author.

## Author contributions

ZB: Conceptualization, Data curation, Formal analysis, Investigation, Methodology, Project administration, Resources, Software, Supervision, Validation, Visualization, Writing – original draft, Writing – review & editing. JD: Conceptualization, Data curation, Formal analysis, Investigation, Methodology, Project administration, Resources, Software, Supervision, Validation, Visualization, Writing – original draft, Writing – review & editing. YZ: Supervision, Writing – review & editing, Investigation, Validation, Visualization. QG: Project administration, Resources, Supervision, Writing – review & editing. RJ: Investigation, Supervision, Validation, Visualization, Writing – review & editing, Conceptualization, Methodology, Project administration, Resources.
