# Corrigendum: Deep learning or radiomics based on CT for predicting the response of gastric cancer to neoadjuvant chemotherapy: a meta-analysis and systematic review

**DOI:** 10.3389/fonc.2024.1433346

**Published:** 2024-05-23

**Authors:** Zhixian Bao, Jie Du, Ya Zheng, Qinghong Guo, Rui Ji

**Affiliations:** ^1^ Department of Gastroenterology, the First Hospital of Lanzhou University, Lanzhou, China; ^2^ Department of Gastroenterology, Xi’an NO.1 Hospital, Xi’an, Shaanxi, China; ^3^ Department of Social Medicine and Health Management, School of Public Health, Lanzhou University, Lanzhou, China; ^4^ Gansu Province Clinical Research Center for Digestive Diseases, The First Hospital of Lanzhou University, Lanzhou, China

**Keywords:** gastric cancer, neoadjuvant chemotherapy, deep learning, radiomics, artificial intelligence, meta-analysis

In the published article, there was an error in affiliations 1,2,3,4. Instead of “Zhixian Bao^1,2†^, Jie Du^3†^, Ya Zheng^2,4^, Qinghong Guo^2,4^, Rui Ji^2,4*^

^1^Department of Gastroenterology, Xi’an NO.1 Hospital, Xi’an, Shaanxi, China.

^2^Department of Gastroenterology, the First Hospital of Lanzhou University, Lanzhou, China.

^3^Department of Social Medicine and Health Management, School of Public Health, Lanzhou University, Lanzhou, China.

^4^Gansu Province Clinical Research Center for Digestive Diseases, The First Hospital of Lanzhou University, Lanzhou, China.”,

it should be “Zhixian Bao^1,2†^, Jie Du^3†^, Ya Zheng^1,4^, Qinghong Guo^1,4^, Rui Ji^1,4*^

^1^Department of Gastroenterology, the First Hospital of Lanzhou University, Lanzhou, China

^2^Department of Gastroenterology, Xi’an NO.1 hospital, Xi’an, Shaanxi, China

^3^Department of Social Medicine and Health Management, School of Public Health, Lanzhou University, Lanzhou, China

^4^Gansu Province Clinical Research Center for Digestive Diseases, The First Hospital of Lanzhou University, Lanzhou, China”.

The authors apologize for this error and state that this does not change the scientific conclusions of the article in any way. The original article has been updated.

