# Supplementary material for: Deep learning or radiomics based on CT for predicting the response of gastric cancer to neoadjuvant chemotherapy: a meta-analysis and systematic review
Source: Front Oncol. 2024 Mar 27;14:1363812. doi: 10.3389/fonc.2024.1363812 (PMC11004479; doi:10.3389/fonc.2024.1363812)
Supplement: Supplementary file 1 [file DataSheet_1.docx]

Supplementary Material

# Supplementary Material-Search strategies

TIME: 2023.09.05

## PubMed

(“Stomach Neoplasms” [Title/Abstract] OR Neoplasm, Stomach[Title/Abstract] OR “Stomach Neoplasm”[Title/Abstract] OR Neoplasms, Stomach[Title/Abstract] OR “Gastric Neoplasms”[Title/Abstract] OR “Gastric Neoplasm”[Title/Abstract] OR Neoplasm, Gastric[Title/Abstract] OR Neoplasms, Gastric[Title/Abstract] OR “Stomach Cancers”[Title/Abstract] OR “Gastric Cancer”[Title/Abstract] OR Cancer, Gastric[Title/Abstract] OR Cancers, Gastric[Title/Abstract] OR Gastric Cancers[Title/Abstract] OR “Stomach Cancer”[Title/Abstract] OR Cancer, Stomach[Title/Abstract] OR Cancers, Stomach[Title/Abstract] OR “Cancer of the Stomach”[Title/Abstract] OR “Stomach Neoplasms”[Mesh]

AND

(“Machine Learning”[Mesh] OR “machine learning”[Title/Abstract] OR “Deep learning”[Title/Abstract] OR “artificial intelligence”[Title/Abstract] OR “Prediction model”[Title/Abstract] OR “Transfer Learning”[Title/Abstract] OR “random forest”[Title/Abstract] OR “artificial neural network” [Title/Abstract] OR ANN[Title/Abstract] OR “Support vector machine”[Title/Abstract] OR SVM[Title/Abstract] OR Nomogram[Title/Abstract] OR XGboost[Title/Abstract] OR Logistic[Title/Abstract] OR Decision tree[Title/Abstract] OR c-index[Title/Abstract] OR ROC[Title/Abstract] OR AUC[Title/Abstract] OR External validation[Title/Abstract]

AND

(“Neoadjuvant Therapy” [Mesh] OR “Neoadjuvant Therapy” [Title/Abstract] OR “Neoadjuvant Therapies” [Title/Abstract] OR “Therapies, Neoadjuvant”[Title/Abstract] OR “Therapy, Neoadjuvant”[Title/Abstract] OR “Neoadjuvant chemotherapy”[Title/Abstract] OR “Neoadjuvant chemotherapies”[Title/Abstract] OR “chemotherapy, Neoadjuvant”[Title/Abstract] OR “Neoadjuvant Treatment”[Title/Abstract] OR “Neoadjuvant Treatments”[Title/Abstract] OR “Treatment, Neoadjuvant”[Title/Abstract] OR “Treatments, Neoadjuvant”[Title/Abstract] OR “Chemotherapy, Adjuvant”[Mesh] OR “adjuvant Therapy” [Title/Abstract] OR “adjuvant Therapies” [Title/Abstract] OR “Therapy, adjuvant” [Title/Abstract] OR “Therapies, adjuvant” [Title/Abstract] OR “adjuvant chemotherapy”[Title/Abstract] OR “adjuvant chemotherapies”[Title/Abstract] OR “chemotherapy, adjuvant”[Title/Abstract] OR “adjuvant treatment”[Title/Abstract] OR “adjuvant treatments”[Title/Abstract] OR “treatment, adjuvant”[Title/Abstract] OR “treatments, adjuvant “[Title/Abstract] OR “Stomach Neoplasms/drug therapy”[Mesh] OR chemotherap*[Title/Abstract] OR Pharmacotherap*[Title/Abstract])

## Cochrane Library

(“Stomach Neoplasms” [Title/Abstract] OR “Stomach Neoplasm” [Title/Abstract] OR “Gastric Neoplasms” [Title/Abstract] OR “Gastric Neoplasm” [Title/Abstract] OR “Cancer of Stomach” [Title/Abstract] OR “Stomach Cancers” [Title/Abstract] OR “Stomach Cancer” [Title/Abstract] OR “Gastric Cancer” [Title/Abstract] OR “Gastric Cancers” [Title/Abstract]) OR (MeSH descriptor: [Stomach Neoplasms] explode all trees OR (Stomach Neoplasms):ti,ab,kw OR (Stomach Neoplasm):ti,ab,kw OR (Gastric Neoplasms):ti,ab,kw OR (Gastric Neoplasm):ti,ab,kw OR (Cancer of Stomach):ti,ab,kw (Word variations have been searched) OR (Stomach Cancers):ti,ab,kw OR (Stomach Cancer):ti,ab,kw OR (Gastric Cancer):ti,ab,kw OR (Gastric Cancers):ti,ab,kw)

AND

(“machine learning”[Title/Abstract] OR “Deep learning”[Title/Abstract] OR “artificial intelligence”[Title/Abstract] OR “Prediction model”[Title/Abstract] OR “Transfer Learning”[Title/Abstract] OR “random forest”[Title/Abstract] OR “artificial neural network”[Title/Abstract] OR “ANN”[Title/Abstract] OR “Support vector machine”[Title/Abstract] OR “SVM”[Title/Abstract] OR Nomogram[Title/Abstract] OR XGboost[Title/Abstract] OR Logistic[Title/Abstract] OR “Decision tree”[Title/Abstract] OR “c-index”[Title/Abstract] OR “ROC”[Title/Abstract] OR “AUC”[Title/Abstract] OR “External validation”[Title/Abstract]) OR (MeSH descriptor: [Machine Learning] explode all trees OR (machine learning):ti,ab,kw OR (Deep learning):ti,ab,kw OR (artificial intelligence):ti,ab,kw OR (Prediction model):ti,ab,kw OR (Transfer Learning):ti,ab,kw OR (random forest):ti,ab,kw OR (“artificial neural network”):ti,ab,kw OR (ANN):ti,ab,kw OR (Support vector machine):ti,ab,kw OR (SVM):ti,ab,kw OR (Nomogram):ti,ab,kw OR (XGboost):ti,ab,kw OR (Logistic):ti,ab,kw OR (“Decision tree”):ti,ab,kw OR (“c-index”):ti,ab,kw OR (ROC):ti,ab,kw OR (AUC):ti,ab,kw OR (“External validation”):ti,ab,kw)

AND

(“Neoadjuvant Therapy” [Title/Abstract] OR “Neoadjuvant Therapies” [Title/Abstract] OR “Neoadjuvant chemotherapy”[Title/Abstract] OR “Neoadjuvant chemotherapies”[Title/Abstract] OR “Neoadjuvant Treatment”[Title/Abstract] OR “Neoadjuvant Treatments”[Title/Abstract] OR “Chemotherapy, Adjuvant”[Mesh] OR “adjuvant Therapy” [Title/Abstract] OR “adjuvant Therapies” [Title/Abstract] OR “adjuvant chemotherapy”[Title/Abstract] OR “adjuvant chemotherapies”[Title/Abstract] OR “adjuvant treatment”[Title/Abstract] OR “adjuvant treatments”[Title/Abstract] OR chemotherap*[Title/Abstract] OR Pharmacotherap*[Title/Abstract]) OR (“Stomach Neoplasms/drug therapy”[Mesh] OR MeSH descriptor: [Chemotherapy, Adjuvant] explode all trees OR (“Neoadjuvant Therapy”):ti,ab,kw OR (“Neoadjuvant Therapies”):ti,ab,kw OR (“Neoadjuvant chemotherapy”):ti,ab,kw OR (“Neoadjuvant chemotherapies”):ti,ab,kw OR (“Neoadjuvant Treatment”):ti,ab,kw OR (“Neoadjuvant Treatments”):ti,ab,kw OR MeSH descriptor: [Neoadjuvant Therapy] explode all trees OR (“adjuvant therapy”):ti,ab,kw OR (“adjuvant Therapies”):ti,ab,kw OR (“adjuvant chemotherapy”):ti,ab,kw OR (“adjuvant chemotherapies”):ti,ab,kw OR (“adjuvant treatment”):ti,ab,kw OR (“adjuvant treatments”):ti,ab,kw OR (chemotherap*):ti,ab,kw OR (Pharmacotherap*):ti,ab,kw OR MeSH descriptor: [Stomach Neoplasms] explode all trees and with qualifier(s): [drug therapy - DT])

## Web of Science

“Stomach Neoplasms” OR “Stomach Neoplasm” OR “Gastric Neoplasms” OR “Gastric Neoplasm” OR “Cancer of Stomach” OR “Stomach Cancers” OR “Stomach Cancer” OR “Gastric Cancer” OR “Gastric Cancers”

AND

“machine learning” OR “Deep learning” OR “artificial intelligence” OR “Prediction model” OR “Transfer Learning” OR “random forest” OR “artificial neural network” OR “ANN” OR “Support vector machine” OR “SVM” OR Nomogram OR XGboost OR Logistic OR “Decision tree” OR “c-index” OR “ROC” OR “AUC” OR “External validation” OR “machine learning” OR “Deep learning” OR “artificial intelligence” OR “Prediction model” OR “Transfer Learning” OR “random forest” OR “artificial neural network” OR “ANN” OR “Support vector machine” OR “SVM” OR Nomogram OR XGboost OR Logistic OR “Decision tree” OR “c-index” OR “ROC” OR “AUC” OR “External validation”

AND

“Neoadjuvant Therapy” OR “Neoadjuvant Therapies” OR “Neoadjuvant chemotherapy” OR “Neoadjuvant chemotherapies” OR “Neoadjuvant Treatment” OR “Neoadjuvant Treatments” OR “adjuvant Therapy” OR “adjuvant Therapies” OR “adjuvant chemotherapy” OR “adjuvant chemotherapies” OR “adjuvant treatment” OR “adjuvant treatments” OR chemotherap* OR Pharmacotherap*

## Embase

(“Stomach tumor” MeSH OR “Stomach Neoplasms” [Title/Abstract] OR “Stomach Neoplasm” [Title/Abstract] OR “Gastric Neoplasms” [Title/Abstract] OR “Gastric Neoplasm” [Title/Abstract] OR “Cancer of Stomach” [Title/Abstract] OR “Stomach Cancers” [Title/Abstract] OR “Stomach Cancer” [Title/Abstract] OR “Gastric Cancer” [Title/Abstract] OR “Gastric Cancers” [Title/Abstract])

AND

(“Machine Learning”[Mesh] OR “machine learning”[Title/Abstract] OR “Deep learning”[Title/Abstract] OR “artificial intelligence”[Title/Abstract] OR “Prediction model”[Title/Abstract] OR “Transfer Learning”[Title/Abstract] OR “random forest”[Title/Abstract] OR “artificial neural network”[Title/Abstract] OR “ANN”[Title/Abstract] OR “Support vector machine”[Title/Abstract] OR “SVM”[Title/Abstract] OR Nomogram[Title/Abstract] OR XGboost[Title/Abstract] OR Logistic[Title/Abstract] OR “Decision tree”[Title/Abstract] OR “c-index”[Title/Abstract] OR “ROC”[Title/Abstract] OR “AUC”[Title/Abstract] OR “External validation”[Title/Abstract])

AND

(“Neoadjuvant Therapy” [Mesh] OR “Neoadjuvant Therapy” [Title/Abstract] OR “Neoadjuvant Therapies” [Title/Abstract] OR “Neoadjuvant chemotherapy”[Title/Abstract] OR “Neoadjuvant chemotherapies”[Title/Abstract] OR “Neoadjuvant Treatment”[Title/Abstract] OR “Neoadjuvant Treatments”[Title/Abstract] OR “ Adjuvant Chemotherapy”[Mesh] OR “adjuvant Therapy” [Title/Abstract] OR “adjuvant Therapies” [Title/Abstract] OR “adjuvant chemotherapy”[Title/Abstract] OR “adjuvant chemotherapies”[Title/Abstract] OR “adjuvant treatment”[Title/Abstract] OR “adjuvant treatments”[Title/Abstract] OR chemotherap*[Title/Abstract] OR Pharmacotherap*[Title/Abstract] OR “Stomach Neoplasms/drug therapy”[Mesh]
